# Farming system context drives the value of deep wheat roots in semi-arid environments

**DOI:** 10.1093/jxb/erw093

**Published:** 2016-03-14

**Authors:** Julianne M. Lilley, John A. Kirkegaard

**Affiliations:** CSIRO Agriculture, GPO Box 1600, Canberra ACT 2601, Australia

**Keywords:** APSIM, deep roots, drought, farming systems, simulation modelling, *Triticum aestivum*, wheat.

## Abstract

The capture of subsoil water by wheat roots can make a valuable contribution to grain yield on deep soils. More extensive root systems can capture more water, but leave the soil in a drier state, potentially limiting water availability to subsequent crops. To evaluate the importance of these legacy effects, a long-term simulation analysis at eight sites in the semi-arid environment of Australia compared the yield of standard wheat cultivars with cultivars that were (i) modified to have root systems which extract more water at depth and/or (ii) sown earlier to increase the duration of the vegetative period and hence rooting depth. We compared simulations with and without annual resetting of soil water to investigate the legacy effects of drier subsoils related to modified root systems. Simulated mean yield benefits from modified root systems declined from 0.1–0.6 t ha^−1^ when annually reset, to 0–0.2 t ha^−1^ in the continuous simulation due to a legacy of drier soils (mean 0–32mm) at subsequent crop sowing. For continuous simulations, predicted yield benefits of >0.2 t ha^−1^ from more extensive root systems were rare (3–10% of years) at sites with shallow soils (<1.0 m), but occurred in 14–44% of years at sites with deeper soils (1.6–2.5 m). Earlier sowing had a larger impact than modified root systems on water uptake (14–31 vs 2–17mm) and mean yield increase (up to 0.7 vs 0–0.2 t ha^−1^) and the benefits occurred on deep and shallow soils and in more years (9–79 vs 3–44%). Increasing the proportion of crops in the sequence which dry the subsoil extensively has implications for the farming system productivity, and the crop sequence must be managed tactically to optimize overall system benefits.

## Highlight

More extensive root systems can capture more water, but leave the soil in a drier state, potentially limiting water availability to subsequent crops.

## Introduction

Several authors have proposed root traits which improve yield in water-limited environments, including increased root elongation rate and depth of rooting (Cooper *et al.*, 1987; Lopes and Reynolds, 2010), root distribution at depth (Hurd, 1968, 1974; O’Brien, 1979), xylem vessel diameter (Richards and Passioura, 1989), angle of seminal roots (Nakamoto and Oyanagi, 1994; Manschadi *et al.*, 2008), and the ratio of root:shoot dry matter (Siddique *et al.*, 1990). Experiments and simulation studies have shown that the capture of subsoil water by deeper wheat roots can make a valuable contribution to yield on a range of deep soil types (Kirkegaard *et al.*, 2007; Lilley and Kirkegaard 2007; Christopher *et al.*, 2008). We briefly describe the evidence for yield benefits from deeper and more extensive root systems, and review several simulation studies estimating the value to the crop of improved capacity to extract water from the soil.

Tennant and Hall (2001) reviewed root depth and water uptake of 20 annual crop and pasture species included in ten different field studies in Western Australia. They concluded that rooting depth was strongly affected by soil type, particularly where limiting conditions occurred and that amelioration of chemical or physical constraints increased root depth. Gregory *et al.* (1984) and Kirkegaard and Lilley (2007) showed that even on potentially deep soils, the depth of soil wetting varies seasonally in the semi-arid zone and that dry soil due to limited rewetting can restrict root depth in some seasons. The root penetration rate (RPR), defined as the rate of downward root growth during the vegetative phase, was suggested as a useful indicator to assess genotypes or management interventions which improve root growth in the field (Kirkegaard and Lilley 2007). A RPR of 1.8mm/^o^C.day was reported by Barraclough and Leigh (1984) for winter wheat growing in unconstrained soil in the UK and twofold differences in RPR between genotypes in container grown plants have been reported (Hurd, 1968). In field soils, maximum RPR of 1.2–1.3mm/^o^C.day have been reported for spring wheats on structured clay soils in Australia (Kirkegaard and Lilley, 2007) and for both spring and winter wheat cultivars grown on sandy soils Denmark (Thorup-Kristensen *et al.*, 2009). Wasson *et al.* (2014) found genetic variation in RPR of 0.9–2.2mm/^o^C.day among a range of Australian and Indian cultivars and a biparental population, although this variation was measured in isolated ‘hill plots’, which do not relate to RPR in field plots (Wasson *et al.*, unpublished). Tennant and Hall (2001) demonstrated that significant increase in water uptake could be achieved by growing longer season crop and pasture species or cultivars. The Danish study of Thorup-Kristensen *et al.* (2009) showed that roots of winter wheat crops grew twice as deep as spring wheat roots due to the longer duration of the crop while the Australian study of Kirkegaard *et al.* (2014) also reported deeper roots and greater water extraction for crops with a longer vegetative period.

Increased root density at depth may result from longer residence time in deeper layers, but genetic differences in root morphology also exist (Gregory, 2006). Field experiments of Christopher *et al.* (2008) and root chamber experiments of Manschadi *et al.* (2008) compared two wheat genotypes varying in root morphology. They found that cv. Seri had a narrower growth angle than cv. Hartog and that the root system of Seri was deeper, denser and more evenly distributed with depth. Christopher *et al.* (2008) concluded that when deep water was present, the genotype with a denser root system (Seri) extracted more soil water, extending the duration of green leaf area and increasing yield. Others have also proposed screening for steeper root angle in other species, to select for deeper root systems which have more effective water capture at depth where root length density typically declines (Manschadi *et al.*, 2010; Lynch 2013). McDonald *et al.* (2012) demonstrated yield benefits for wheat cultivars with a narrow seminal root angle in a range of Australian environments. Genotypic variation in the vigour of spring wheat root systems has also been demonstrated by Richards *et al.* (2007) and Palta and Watt (2009), and more recently considerable effort has been invested in screening large numbers of wheat lines in Australia in search of deeper and more extensive root systems (Wasson *et al.*, 2012, 2014). However, White and Kirkegaard (2010) showed that in southern Australian soils, roots in subsoils are often clumped in soil pores and channels with poor root-soil contact, limiting soil water extraction. To increase water extraction from depth, roots must overcome these constraints and explore a greater soil volume. Field measurements of increased water extraction are important, validating the assumption of the benefits of greater root proliferation, although this validation needs to be site specific.

The usefulness of individual root traits is largely determined by the pattern of water availability in the target environment. As a consequence, interactions between these root traits and the seasonal rainfall distribution, soil type and crop management at specific sites influence their impact on yield (Chenu *et al.*, 2011, 2013).

The advantages of timely sowing for improved water-use efficiency and yield of cereals in rain-fed environments are widely known (French and Schultz, 1984; Stapper and Fischer 1990; Hocking and Stapper 2001), but recently recommended sowing times have been re-evaluated in different regions in the face of climate, equipment and varietal changes (Kirkegaard *et al.* 2014). In southern Australia, there has been a decrease in autumn rains on which the wheat crop was traditionally sown and a drier and hotter spring, while summer rainfall has been stable (Pook *et al.*, 2009; Cai *et al.*, 2012). This has stimulated the development of earlier sowing systems based on improved summer fallow management practices to increase soil water storage and use of slower maturing varieties at lower density to maintain optimum flowering times while increasing yield potential. (Kirkegaard and Hunt 2010; Hunt *et al.*, 2013; Kirkegaard *et al.*, 2014; Richards *et al.*, 2014). The presence of stored soil water increases the likelihood of good crop establishment (Hunt and Kirkegaard, 2011) and the longer vegetative period of the slow-maturing cultivars increased rooting depth and access to stored water during grain-filling (Richards *et al.*, 2014; Kirkegaard *et al.*, 2014). Consequently, there has been a significant transition to earlier sowing of wheat in southern Australia (Kirkegaard *et al.*, 2014), but it is likely that much of the benefit may rely on the availability of deep stored water, which will vary from season to season.

Conclusions drawn from field experiments are limited to the range of seasons experienced, so simulation studies are often used to extrapolate across more seasons. In addition, cultivars that differ in root traits may also differ in shoot traits, confounding the experimental evidence for benefits of variation in root vigour. For example, the stay-green trait in sorghum (*Sorghum bicolor*) has been related to yield benefits in dry conditions but was also associated with canopy development, leaf anatomy, extensive root growth and greater water uptake (Borrell *et al.*, 2014). Studies in wheat found that expression of the stay-green trait was associated with a yield benefit but was dependant on availability of deep soil water (Christopher *et al.*, 2008). Simulation studies offer the opportunity to hypothetically modify genetic characteristics of root systems without modifying shoot systems. Several studies which used simulation analysis to investigate the benefits to crop yield of modified root systems are summarized in Table 1. The extent to which the simulation studies have been validated in the field varies. The model of King *et al.* (2003) is conceptual while the others are process-based and have been validated in linked field studies to various degrees.

**Table 1.** Summary of published studies simulating the effects of modified root growth on wheat yield

Author	Location^a^	Model	Frequency of soil water resetting	Soil water set point	Trait modified	Other factors simulated	Yield benefit
Dreccer *et al.* (2002)	VIC (2 sites)	LINTULCC2	Annual at sowing	95% of PAW	Max. root depth RLD Root uptake rate	Shallow soil (0.9 and 1.1 m)	Up to 16.5%
King *et al.* (2003)	UK	Not stated	Annual at anthesis	Not stated	Root distribution within profile	Investigation of capture of water and N in profile during grain-filling	Not reported
Manschadi *et al.* (2006)	Southern QLD (3 sites)	APSIM	Annual at sowing	1/3 PAW, 2/3 PAW, full	Greater efficiency of water uptake below 70cm	Capturing root angle	14.5% in dry seasons
Lilley and Kirkegaard (2007)	Southern NSW (3 sites)	APSIM	Annual at previous harvest	After crop or lucerne	Root depth limited to 1.2 m or unlimited	Following annual crop or lucerne	Subsoil water worth 35kg/ ha.mm
Wong and Asseng (2007)	WA 2 sites	APSIM	Annual prior to sowing rain	Dry or 30mm stored	None modified	Two soil types; 18 levels of subsoil constraint (compaction)	Varied with season, related to rainfall
Semenov *et al.* (2009)	UK, Spain	Sirius	Annual at sowing	Fully wet	Rate of descent, exploration	Two soil depths (0.75 and 1.5 m)	70% in dry years
Farre *et al.* (2010)	WA 30 sites	APSIM	Annual on 1 Jan	Lower limit of extraction	None modified	Removal of subsoil constraint (compaction in 20–40cm layer)	0–2.5 t ha^−1^ Related to seasonal rainfall and soil type
Lilley and Kirkegaard (2011)	WA, NSW, QLD (5 sites)	APSIM	Annual at previous harvest	After crop or lucerne	Rate root descent Efficiency of uptake	Sowing date; prior management	Mean 0.3–0.4 t ha^−1^

^a^Australian states listed are: QLD, Queensland; NSW, New South Wales; VIC, Victoria; WA, Western Australia.

Farre *et al.* (2010) and Wong and Asseng (2007) investigated the removal of subsoil constraints at over 30 locations across Western Australia, allowing increased root growth and greater access to soil resources. In that environment, the yield benefit was strongly related to the severity of the constraint and seasonal rainfall, as low rainfall years caused incomplete soil wetting. Benefits of constraint removal were much smaller (<1.0 t ha^−1^) on duplex soils where rooting depth was restricted to 0.9 m compared to the sandy soil (rooting depth 1.5–1.8 m), where yield benefits of up to 2.5 t ha^−1^ were predicted (Farre *et al.*, 2010).

King *et al.* (2003) used a model that described size and distribution of winter wheat root systems at anthesis. They investigated the predicted impact of a change in root system characteristics such as root distribution with depth, proportional dry matter partitioning to roots, resource capture coefficients for water and N capture and grain yield of cereal crops in the UK. They concluded that a larger investment by the crop in fine roots at depth in the soil, and less proliferation of roots in surface layers would improve yields by accessing extra resources.

Dreccer *et al.* (2002) investigated the impact of ±2% or ±5% change in several root traits including maximum depth of extraction, root length density distribution with depth, and maximum rate of water uptake per unit length. Their simulation was targeted to shallow soils (0.9–1.1 m) in a low-rainfall area of Victoria, Australia and demonstrated up to 16.5% yield benefit from greater rooting depth and a smaller effect of improved rate of water uptake (efficiency; 2.5%). Semenov *et al.* (2009) also simulated rate of root descent and efficiency of water uptake on shallow (0.75 m) soils in UK and Spain as well as on deeper soils (1.5 m). They found that doubling of RPR had no impact on yield in either Spain or the UK, although slowing RPR decreased yield. Similarly, increased efficiency of water extraction produced a small (1.1%) increase in yield. The authors attributed the small response to the limited soil depth (0.6–0.75 m). The studies of Dreccer *et al.* (2002), King *et al.* (2003) and Semenov *et al.* (2009) all initialized simulations with a full soil water profile, and in these situations soil water content did not limit root penetration. While appropriate to the higher rainfall environments, profile water content at sowing is highly variable in many semi-arid environments and in many Australian examples, profiles do not fully rewet (Kirkegaard and Lilley 2007; Lilley and Kirkegaard 2007, 2011; Wong and Asseng 2007; Farre *et al.*, 2010), which limits root depth.

Manschadi *et al.* (2006) investigated modification of root distribution in the soil profile in Queensland, Australia, replicating characteristics of two wheat cultivars (Hartog and Seri) which differed in root density distribution. Their simulations were also reset at sowing in each year with a range of starting soil water conditions (total available water content: 130, 185 or 300mm depending on location. At each location the profile was set at 1/3, 2/3 capacity or full at sowing). In those summer-dominant rainfall environments the crop relied to a large extent on stored water rather than in-crop rainfall, so the impact of initial conditions was significant. Mean yield increased and year-to-year variability decreased as initial soil water content increased, while the relative benefit of the more extensive root system decreased with increasing initial soil water content.

All of the studies mentioned above demonstrated that on deeper soils with a plentiful initial soil water supply, increased root density, uptake efficiency or root depth led to predicted increases in water uptake and grain yields. On shallow soils (~1 m), predicted yield differences were small in the study of Semenov *et al.* (2009), but up to 16.5% in the study of Dreccer *et al.* (2002).

Lilley and Kirkegaard (2011) conducted simulation analyses in Australia investigating the interaction of agronomic management with root modification on deep soils. They showed that in many years, fallow rainfall and in-crop rainfall were insufficient to fully wet the profile and final root depth of the subsequent crop was restricted by dry soil layers. The study showed that increased capture of deep water can occur through selection of cultivars with more extensive (faster descent and more effective) root systems. However, the impact of individual root traits on grain yield varied with site and season and interacted strongly with crop management, antecedent soil water content, seasonal rainfall distribution and soil type. Although this study considered the impacts of previous management and fallow rainfall conditions by resetting the soil water at the previous harvest (15 December) rather than at sowing, the simulations were restricted to single years. In reality, more effective root systems will leave the soil in a drier state, potentially leaving a legacy of limited water availability to subsequent crops and diminishing the overall system benefit of deeper roots.

The analysis of Lilley and Kirkegaard (2011) was also restricted to soils of at least 1.6 m depth, where deep and effective root systems will have the greatest benefits. However, much of the Australian cropping zone has inhospitable subsoils below 0.5–1.0 m (saline, sodic, too acid, too alkaline, too high in boron, aluminium or manganese, or too low in zinc) and other nutrients that roots need (Passioura, 2002; Nuttall *et al.*, 2003; Adcock *et al.*, 2007; Nuttall and Armstrong, 2010). As a result, the previous simulation studies may be overestimating the value of modified root systems for many Australian cropping soils. For example, sodicity constraints have been reported for 59% of Victorian and 63% of South Australian arable land (Ford *et al.*, 1993) and are estimated to affect more than 26% of Queensland (Dang *et al.*, 2006; MacEwan *et al.*, 2010) and around 50% of arable land nationally.

Since the majority of previous studies used full soil water profiles at sowing, annual resetting and/or deep soils in the analysis of the value of deep roots, it is possible there has been an overestimation of the likely benefits of deep roots at the systems scale. To investigate this possibility, we conducted a simulation analysis to investigate the impacts of annual resetting of soil water content vs continuous simulation to capture the legacy effect on the predicted value of modified root systems, using diverse semi-arid environments in Australia as a case study. We also compared the benefits for crop yield of modified root systems with those of earlier sowing, an agronomic intervention known to increase maximum rooting depth (Kirkegaard and Lilley 2007; Thorup-Kristensen *et al.*, 2009; Kirkegaard *et al.*, 2014), and the trajectory of shoot biomass and water demand in the crop. Finally, we considered the importance of soil depth, given that previous work suggested benefits of modified root systems would be limited on shallow soils (Tennant and Hall, 2001; Wong and Asseng, 2007; Semenov *et al.*, 2009; Farre *et al.*, 2010; McDonald *et al.*, 2012).

While this review focuses on increasing yield through the increased capture of water, we recognize that more extensive root systems will also capture other resources such as N and other nutrients. We have maintained N at non-limiting levels throughout our study to avoid confounding effects on N cycling. The wider implications for modified root systems within wheat farming systems in the context of deep water and N use is considered in Thorup-Kristensen and Kirkegaard (2016).

## Methods

Simulations were conducted to represent a continuous cropping sequence at eight locations in Australia, varying in climate, soil type and soil depth. Three factors were varied at each site, which are summarized in Table 2 and described in detail in the sections below. Soil water content in the simulations was either reset annually after harvest to represent a typical soil profile following an annual crop (similar to Lilley and Kirkegaard, 2011), or allowed to run continuously, capturing the soil water profile left by the previous annual crop (as in Lilley *et al.*, 2004). This comparison was made because in Australia the soil often does not refill between cropping seasons and so legacies of drier soil can persist, especially when the subsoil is dry. The analysis compared the yield of standard wheat cultivars with (i) cultivars modified to have a faster rate of downward root growth and increased water extraction efficiency in the subsoil (>0.6 m), (ii) slower-maturing cultivars sown 3 weeks earlier and (iii) a combination of (i) and (ii) (Table 2).

**Table 2.** Summary of the site, crop management and root modification factors included in the factorial simulation analysis

Factors	No. levels	Treatments	
**Site** ^a^	8	Dalby, QLD; Harden, NSW; Cootamundra, NSW; Ardlethan, NSW; Birchip, VIC; Paskeville, SA; Esperance, WA; Wongan Hills, WA	
**Soil water setting**			
Legacy effect	2	(i) Annual reset of soil water to represent profile following typical previous annual crop.^b^ (ii) No resetting, soil water content dependant on extraction by previous wheat crop.	
**Genetic**			
Root systems	2	(i) Standard (ii) Modified – fast (+20%) and more efficient below 0.6m	
**Crop management**			
Sowing window	2	(i) Early	(ii) Conventional
Cultivar		Slow-developing cultivar (e.g. Bolac, Lancer)	Mid-fast developing cultivar (e.g. Mace, Scout, Spitfire)
Date range of sowing window		QLD: 5 May–28 May NSW: 19 April–9 May VIC: 10 April–30 Apr SA: 15 April–7 May WA: 12 April–30 Apr	QLD: 29 May–21 June NSW: 10 May–30 May VIC: 1 May–30 May SA: 8 May–30 May WA: 1 May–30 May
Sowing rule^c^		>15mm over a 7-d period 0–20cm soil layer, > 50% PAW In WA: 0–0.4 m, >15mm PAW In QLD: 0–2.2 m, >100mm PAW	>15mm over a 10-d period 0–10cm soil layer, >50% PAW In WA: 0–0.4 m, >15mm PAW In QLD: 0–2.2 m, >100mm PAW

^a^ Australian states listed are: QLD, Queensland; NSW, New South Wales; SA, South Australia; VIC, Victoria; WA, Western Australia.

^b^ Reset dates for each site are listed in Table 2 and soil water content is shown in Fig. 1.

^c^ OR sown into dry soil at the end of the window if the criteria were not met.

Wheat crops were simulated with APSIM-Wheat (the Agricultural Production Systems SIMulator, version 7.7 (Holzworth *et al.*, 2014; http://www.apsim.info), using Soilwat as the water balance module.

### Site descriptions

The eight sites selected represented three contrasting climatic zones of the Australian wheat belt: (i) temperate with equi-seasonal rainfall distribution; (ii) Mediterranean (winter-dominant rainfall); and (iii) a subtropical environment with summer-dominant rainfall (Table 3). Five of the sites were those selected in the study of Lilley and Kirkegaard (2011), and three further sites were added in the Mediterranean zone. The additional sites all had soils with a maximum rooting depth for annual crops of ~1 m due to chemical and physical subsoil constraints.

**Table 3.** Key climatic variables and soil characteristics and location information for the eight sites included in the simulation study. Date of resetting for annually reset simulations (the date of the latest harvest at the site in 100 years) is also shown

Climate/ location^a^	Latitude, Longitude	Rainfall (mm)		Australian Soil Classification (Isbell, 2002)	Surface soil (0–0.1 m)		Soil (0.1–1.0 m)		Soil below 1 m		Potential root depth (m)	PAW (mm)	
		Annual mean (range)	April–Oct. mean (range)		pH (in water)	Bulk density (g cm^−3^)	pH (in water)	Bulk density (g cm^−3^)	pH (in water)	Bulk density (g cm^−3^)			Reset Date
*Equi-seasonal/temperate*													
Harden, NSW	−34.56, 148.37	605 (202–1104)	368 (117–676)	Red Chromosol	5.8	1.35	7.0	1.70	8.0	1.80	1.6	171	14 Dec.
Cootamundra, NSW	−34.64, 148.02	619 (204–1156)	384 (127–765)	Red Kandosol	5.8	1.45	7.0	1.57	8.0	1.65	2.2	228	11 Dec.
Ardlethan, NSW	−34.36, 146.90	471 (174–864)	284 (85–551)	Red Kandosol	5.8	1.45	7.0	1.57	8.0	1.65	2.2	228	27 Nov.
*Winter-dominant/Mediterranean*													
Birchip, VIC	−35.98, 142.92	365 (111–729)	246 (62–440)	Hypercalcic Calcarosol	8.6	1.33	9.6	1.41	9.3	1.41	0.9	114	12 Dec.
Paskeville, SA	−34.04, 137.90	602 (262–1063)	330 (101–645)	Hypercalcic Calcarosol	8.2	1.30	8.5	1.50	9.2	1.60	1.0	90	29 Nov.
Wongan Hills, WA	−30.84, 116.73	369 (144–672)	302 (112–518)	Yellow-Orthic Tenosol (deep sand)	5.8	1.39	5.2	1.56	5.5	1.55	2.5	153	20 Nov.
Esperance, WA	−33.60, 121.78	517 (260–793)	390 (210–581)	Chromosol (Sand over clay duplex)	5.3	1.40	5.8	1.50	5.0	1.50	1.0	68	19 Nov.
*Summer dominant/subtropical*													
Dalby, QLD	−27.18, 151.26	665 (333–1043)	268 (73–610)	Black Vertosol (heavy clay)	8.4	1.10	8.8	1.12	9.2	1.20	2.2	371	7 Nov.

^a^ Australian states listed are: QLD, Queensland; NSW, New South Wales; SA, South Australia; VIC, Victoria; WA, Western Australia.

### Soil description

**Fig. 1.**
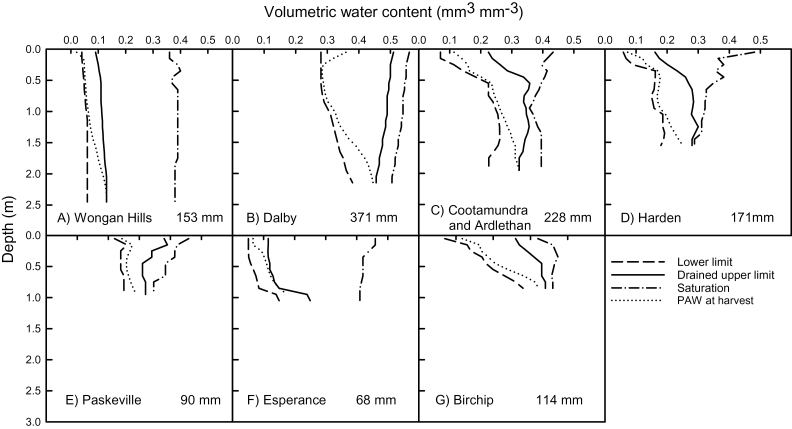
Volumetric water content of the soils at the eight sites at saturation, drained upper limit (DUL), lower limit (LL) of plant water extraction and plant available water content (PAW-harvest) (to which annual simulations were reset – see Table 2) are shown in panels A–G). PAW-harvest is the median PAW at harvest from 100 years of continuous simulation of a cultivar with standard roots and conventional sowing date. Ardlethan and Cootamundra are represented by the same soil. Source of soil water characterization can be found in Supplementary Table 1.

Details of the soils for each of the eight sites are summarized in Table 3. Soils were parameterized using measured soil data at each site in 0.1 m layers to the depths indicated. Soil characteristics were obtained from soil measurements, or extracted from the ApSoil database (https://www.apsim.info/Products/APSoil.aspx) and full details of APSIM parameters for each soil are included in Supplementary Table 1. Volumetric water content at saturation, drained upper limit (DUL), and lower limit of crop extraction (LL) for each of the soil types at the eight sites are shown in Fig. 1. Soil water content at saturation was determined from measured bulk density values, DUL was determined from field measurements of fully wet then drained profiles (Hochman *et al.*, 2001), and LL from field measurements described below. At Harden, maximum root depth was limited to 1.6 m by a weathered granite layer in the soil. At Dalby, downward root growth rate was slowed below 1.6 m by subsoil salinity. A combination of high pH, high chloride and boron concentrations throughout the profile of the Hypercalcic Calcarosol at Birchip resulted in poor soil exploration and constrained roots to a maximum depth of 0.9 m, while at Paskeville high boron content (>30mg kg^−1^) below 1 m constrained maximum root depth to 1.0 m. Rooting depth of the duplex soil at Esperance was constrained by soil acidity (pH of 5.0 below 1.0 m), physical properties which limited water infiltration, and gravel at 1 m depth. APSIM-Wheat accurately simulates wheat yields across a broad range of environments in Australia and it has been carefully validated on Red loam soils (Kandosols and Chromosols; Isbell, 2002) in southern NSW (Lilley *et al.*, 2004; Lilley and Kirkegaard, 2007). Those studies involved detailed comparison of simulation outputs with experimental data for biomass growth, grain yield and soil water dynamics to establish confidence in the capacity of the APSIM-Wheat model to simulate the processes involved in this analysis. For the other soils, the model has been well validated on similar soil types to those used in this study. These include deep sands (Tenosols) and sand over clay duplexes (Chromosols) in WA (Lawes *et al.*, 2009; Oliver and Robertson, 2009) and deep clays (Vertosols) in northern Australia (Hochman et al., 2001, 2007; Wang *et al.*, 2003), and calcareous soils with subsoil constraints below 1 m (Calcarosols) in southern Australia (Rodriguez *et al.*, 2006; Hochman *et al.*, 2009, Hunt *et al.*, 2013).

### Simulation treatments – accounting for the soil water legacy

In all simulations the crop sequence was assumed to involve continuous cropping of productive annual crops such as wheat, barley or canola, and water extraction patterns by these annual crops are generally similar to wheat. In this case, for simplicity of the analysis we simulated continuous cropping sequences with wheat sown every year as a representative annual crop. Soil water content at sowing was simulated in two ways:

(i) *Annual reset.* Similar to the method of Lilley and Kirkegaard (2011) the soil water profile was reset annually, on the latest predicted harvest date of all crops in the 100-year simulation at each site. Reset date ranged from 7 November at Dalby to 14 December at Harden (Table 3). The soil water content was reset to the median profile at harvest over 100 years of continuous simulation and is shown in Fig. 1. These simulations were run as single years commencing on the reset date. This differed from the study of Lilley and Kirkegaard (2011) who reset on 15 December each year to a profile which was deemed to represent an annual crop (at the LL from 0 to 1.2 m, below which soil was at the DUL). The change was made as a 15 December reset date was not appropriate for all sites (up to 5 weeks after harvest of the previous crop) and to more accurately represent the soil profile at harvest as these previous rules did not fit the shallow soils. A sensitivity analysis showed that the previous setting by Lilley and Kirkegaard (2011) produced similar results on the deep soil sites, except at Dalby where resetting occurred 5 weeks earlier, and the median profile was drier than a profile that was dry to 1.2 m and full from 1.2 to 2.5 m. The soil water content at sowing was simulated as a consequence of soil water content on the reset date and subsequent rainfall and evaporation until sowing, assuming the summer fallow was maintained weed-free with stubble retained.
(ii) *Continuous*. Simulations were run continuously with a wheat crop sown every year from 1900 so that soil water content at sowing in each year was related to the previous long-term cropping history as well as seasonal rainfall and evaporation. Thus for a continuous simulation using wheat with a root system modified to extract water more effectively below 0.6 m, the improved drying of the subsoil every year can compound as a legacy unless there is adequate rainfall to fully recharge the profile. Therefore, plant available water (PAW) at sowing differed from that in the annually reset simulations. The simulations were run for the years 1900 to 2014 of the climatic record, with the first 15 years discarded so that the effect of initial soil water profile was replaced by the legacy of the crops in the first 15 years.

### Simulation treatments – root modification

To investigate potential impacts of genetic modifications to roots on wheat productivity, root characteristics were modified following the method of Lilley and Kirkegaard (2011). Our earlier study considered rate of root descent and increased water extraction efficiency (i.e. a greater potential rate of water extraction) separately, since they are considered distinct targets for breeding. That study showed that the benefit of each component depended on site conditions (soil type and climate), however in general the benefit of more efficient water extraction was greater than that of faster root descent. The benefits were generally additive and in this analysis we consider the combined effect.

APSIM-Wheat uses a maximum root penetration rate (RPR) for field grown wheat of 1.2mm/^o^C.day up to the start of grain filling (for daily average temperatures up to 25^o^C) (Wang and Smith, 2004). To represent the effect of soil drying on soil strength and root growth, the RPR through a soil layer is reduced at low water content. RPR is unaffected by soil water content until the proportion of PAW falls below 25%. Below 25% PAW, the RPR is reduced linearly from the maximum RPR to zero root downward growth when no PAW remains. In the modified treatment we configured APSIM-Wheat to increase the rate of root descent by 20% (i.e. maximum RPR 1.44mm/^o^C.day) as simulated in Lilley and Kirkegaard (2011) and within the range reported for field grown plants (maximum 2.2mm/^o^C.day; Wasson *et al.*, 2014).

**Fig. 2.**
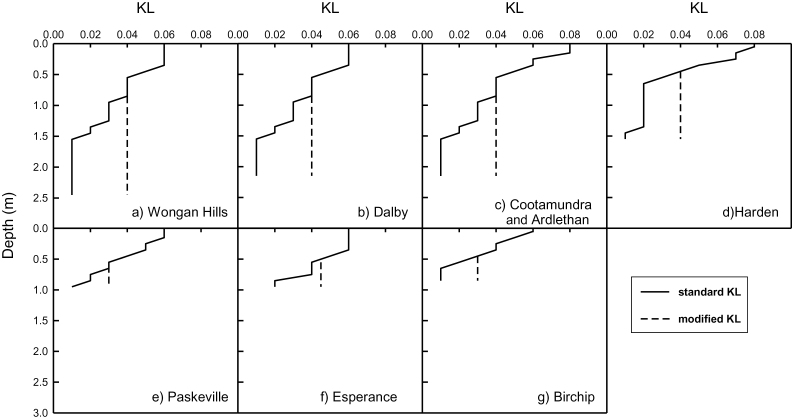
Water extraction efficiency (KL) for each layer for the seven soil types used in the simulations. Extraction efficiency is shown for standard root systems (solid line) and for the modified root systems (dashed line). Extraction efficiency represents the maximum proportion of PAW that can be extracted from each layer each day.

The capacity of wheat root systems to extract water from the soil decreases with depth, due to reduced root length density, increased clumping and confinement of roots to pores and structural features of the soil, and reduced root-soil contact. The APSIM model captures this effect with the KL parameter (Wang and Smith, 2004). The KL value of each soil layer is the maximum proportion of PAW remaining in the soil that can be extracted from the layer on any day, and is set empirically to fit observed data for each combination of crop and soil type (Meinke *et al.*, 1993, Robertson *et al.*, 1993; Dardanelli *et al.*, 2003). The actual volume of water extracted from a layer is limited by the crop demand, which is met preferentially from upper most layers first, and the presence of roots in the layer. The robustness and limitations of this approach have been discussed previously (Wang and Smith, 2004; Manschadi *et al.*, 2006). The standard KL profile fitted to observed rates of water extraction by existing wheat varieties for each soil type is shown in Fig. 2. For the modified root system, we increased the extraction efficiency (potential rate of water extraction) of wheat roots in the subsoil by maintaining the KL values at those observed at 0.6 m. As a consequence, the capacity to extract water from the subsoil below 0.6 m was 30–50% of that in the surface, rather than 10–20% as is commonly measured in current wheat varieties.

### Simulation treatments – sowing window

In order to investigate previously demonstrated advantages of earlier sowing for deeper rooting and water extraction we simulated the conventional sowing window at each site, along with a window which opened 3 weeks earlier (Table 3). For the conventional sowing, a mid-fast developing wheat cultivar (e.g. Mace, Scout, Spitfire) was sown, while in the earlier sowing window a slow-developing cultivar (e.g. Bolac, Lancer) was sown. APSIM phenology parameters, *vern_sens* and *photop_sens* were 2.3 and 3.9, respectively, for the slow-developing cultivar and 0.5 and 3.0, respectively, for the mid-fast developing cultivar. In each year, sowing occurred within the prescribed window as soon as sowing criteria described in Table 2 were met. Criteria consisted of a minimum rainfall within a set period as well as minimum soil water content in upper profile layers (Table 2). If the criteria were not met within the sowing window, the crop was sown into dry soil on the last day of the window, and emergence occurred after the next rainfall event. Simulated anthesis and maturity dates of these cultivars matched that of local well-adapted cultivars at each site. These cultivars flowered in the optimal windows in each environment and mean anthesis and maturity dates of the standard and the early-sown cultivars occurred within 2 d.

### Simulation details

For all sites, daily climatic data (rainfall, solar radiation, pan evaporation, maximum and minimum temperatures) were extracted from the SILO Patched Point Dataset (Jeffrey *et al.*, 2001; http://www.bom.gov.au/silo/). Climatic information is summarized in Table 3.

Soil N in the simulations was maintained at levels non-limiting to plant growth. Fertilizer was applied at sowing and 40 d after sowing so that soil mineral N content was 200kg N/ha at the sites with deep soil (Harden, Cootamundra, Ardlethan, Wongan Hills and Dalby) and 150kg N/ha at the sites with shallow soil (Birchip, Paskeville, Esperance).

Factorial combinations of the treatments in Table 2 produced eight simulation runs at each of the eight sites, a total of 64 site × soil water legacy × sowing window × root modification runs over a 100-year period. A range of simulation outputs were compiled to provide insights into the magnitude and mechanism of yield benefits arising from differences in root systems associated with either differences in soil water resetting, agronomic management (early sowing) or hypothetical genetic modification (more effective roots). The data extracted from the simulation runs included the soil water content at sowing, final rooting depth, total and distribution of water uptake from the soil profile, flowering date and grain yield. In general, to compare the three treatment factors, differences between treatments were calculated within each year for each variable. The conventionally sown, standard root system cultivar was used as the reference and a set of differences between treatments within reset simulations and within continuous simulations were calculated. The range, mean or median of the within-year differences were calculated for each site, rather than comparisons between long-term means for each scenario.

## Results

### Rooting depth

Simulated final rooting depths on deeper soils of the standard cultivar at a conventional sowing date (Table 4) were similar to those reported previously by Lilley and Kirkegaard (2011) and experimentally by others at those sites (Forrest *et al.*, 1985; Hamblin and Tennant, 1987; Kirkegaard *et al.*, 2007; Milroy *et al.*, 2008). On the shallower constrained soils, the roots usually reached the bottom of the profile (1.0 m) at Paskeville and always at Esperance, while at Birchip impediments to root growth such as dry soil and chemical constraints resulted in an average rooting depth of 0.7 m. These results on shallower soils are similar to experimental results reported by Tennant and Hall (2001), Dreccer *et al.* (2002), Rodriguez *et al.* (2006), Oliver and Robertson (2009), and Hunt *et al.* (2013). Use of annual resetting or continuous simulation made little difference to final rooting depth, however variability was greater on deep soils in the continuous simulation (data not shown).

**Table 4.** Mean and range of rooting depth at maturity of wheat crops (standard cultivar, conventional sowing date) for 100 years of continuous and annually reset simulations at eight sitesMean extra final root depth and difference in water uptake (total and post-anthesis) achieved by simulating cultivars with modified root systems (Mod) and/or earlier sowing is also shown.

Location^a^	Final root depth (m) – standard cultivar, conventional sowing			Mean extra root depth (m)				Mean extra water uptake (mm)				Mean extra post-anthesis uptake (mm)			
	Reset	Continuous		Reset	Continuous			Reset	Continuous			Reset	Continuous		
	Mean	Mean	Range	Mod	Mod	Early	Early, mod	Mod	Mod	Early	Early, mod	Mod	Mod	Early	Early, mod
Wongan Hills, WA	1.98	1.96	0.96–2.02	0.34	0.17	0.25	0.18	29	17	29	39	21	11	2	7
Dalby, QLD	1.46	1.44	0.18–1.76	0.23	−0.06	0.03	−0.07	15	3	14	16	9	−1	−10	−11
Cootamundra, NSW	1.77	1.76	0.83–1.88	0.31	0.26	0.27	0.32	19	16	31	45	15	12	−9	0
Ardlethan, NSW	1.52	1.59	0.44–1.88	0.25	0.13	0.15	0.13	14	10	26	33	11	8	−10	−6
Harden, NSW	1.60	1.59	0.98–1.60	0.00	0.00	0.00	−0.01	9	7	26	33	4	3	−11	−8
Paskeville, SA	0.97	0.97	0.71–0.98	0.03	0.02	0.02	0.02	4	2	22	24	2	1	−7	−6
Esperance, WA	1.00	1.00	0.87–1.00	0.00	0.00	0.00	0.00	4	4	26	30	1	1	−5	−4
Birchip, VIC	0.70	0.67	0.24–0.72	0.04	0.02	0.03	0.06	4	3	24	27	2	1	−5	−3

^a^ Australian states listed are: QLD, Queensland; NSW, New South Wales; VIC, Victoria; SA, South Australia; WA, Western Australia.

**Fig. 3.**
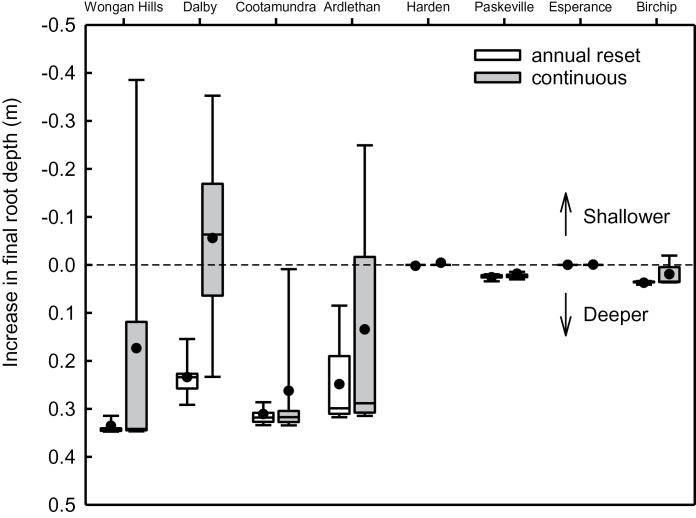
Box plots of simulated change in final root depth of modified root systems relative to the standard cultivar at eight sites varying in climate and soil type. Simulations were either reset annually (white) or run continuously. Median (solid line), mean (circle), 25th and 75th percentile (box), 10th and 90th percentile (whisker) are presented for 100 years of simulation.

In simulations where roots were modified (downward growth 20% faster), the mean benefit to rooting depth on deep soils was smaller in continuous simulations (−0.06–0.26 m) than in the reset simulations (0.23–0.34 m; Table 4). In addition, variability was greater in the continuous simulation (Fig. 3) as root depth was more frequently restricted due to soil drying by the previous crop. At Dalby, root modification resulted in slightly shallower mean root depth in the continuous simulation, due to reduced root penetration in dry soil. On soils with a depth constraint (Harden, Paskeville, Esperance and Birchip) there was no effect of modified root systems on final root depth, and roots simply reached the bottom of the accessible profile sooner.

Mean final root depth of early-sown crops was increased by 0.03–0.27 m on unrestricted soils, compared to crops sown on the conventional date (Table 4). This was due to an approximately 3-week longer vegetative period when downward root growth occurs. Earlier sowing of the slow-developing cultivars, which also had modified root systems, resulted in a small further increase in the mean root depth at Cootamundra, no effect at Ardlethan, and shallower roots at Wongan Hills and Dalby (Table 4). In the shallow soils at Paskeville and Birchip there were very small (0.02–0.03 m) increases in root depth of early-sown cultivars, but not at Harden or Esperance where roots reached the bottom of the profile when sown in the conventional window.

### Water uptake

**Fig. 4.**
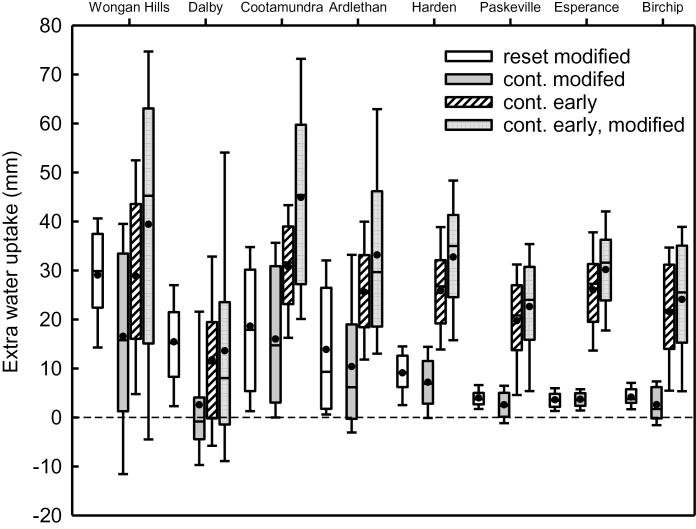
Box plots of simulated extra water uptake (mm) of cultivars with either modified root systems and/or were sown early relative to the standard cultivar sown in the conventional window at eight sites varying in climate and soil type. Simulations were either reset annually (white) or run continuously (shaded) so that the legacy of crop history affected soil water content. Median (black line), mean (circle), 25th and 75th percentile (box), 10th and 90th percentile (whisker) are presented for 100 years of simulation.

More rapid root descent and increased final root depth, combined with more efficient water uptake below 0.6 m resulted in a greater average crop water uptake for crops with modified root systems (Table 4). A smaller water extraction advantage was evident in the continuous simulation compared with the annual reset at all sites (Table 4). Modified root systems led to an average 7–17mm of additional water extraction on deeper soils, and 2–4mm on shallow soils (Table 4). The difference in water uptake was highly variable across seasons, ranging from a reduction of 18mm to an increase of 44mm, with greatest variability seen on deeper soils (Fig. 4). For earlier-sown crops, mean additional water uptake was 14–31mm greater than for conventionally sown crops (Table 4) due to both deeper roots associated with longer duration of root descent, and a longer duration of the period of water extraction. Notably, the effect of early sowing on extra water uptake was relatively similar on deep and shallow soils (Table 4). The effects of early sowing and modified root systems were largely additive, with the combination increasing water uptake (mean: 16–45mm; Table 4; Fig. 4). The uptake at Dalby for modified and/or early-sown crops was significantly less than for other sites with deep soils. Variability in uptake was generally greater for modified than standard root systems on all soils (Fig. 4). Although mean uptake was higher for early-sown crops on deep soils, variability was similar, but increased when the root system was modified as well. On shallow soils, the larger variability predicted for early sowing was associated with much greater mean extra uptake (Fig 4.).

An analysis of the timing of water uptake showed that where root systems were modified, around two-thirds of the additional water extraction occurred post-anthesis, except at Dalby where extra post-anthesis extraction was small (Table 4). When the crop was sown early at all sites except Wongan Hills, post-anthesis extraction was smaller (mean reduction 5–11mm; Table 4). The increase in total water uptake for early-sown crops was due to much greater pre-anthesis uptake, creating a drier soil by anthesis and less water was available for post-anthesis uptake.

### PAW at sowing (soil water legacy effect)

In the annually reset simulations, PAW at sowing varied across the sites according to soil water holding capacity, and fallow rainfall (Table 5). For simulations of standard cultivars, the mean PAW at sowing was similar in the reset and continuous simulations. However, the variability was much greater in continuously run simulations, because the soil water content was also affected by water extraction of the previous crops (data not shown). For continuous simulations, modified root systems led to reduced PAW at sowing for deep soils (mean 17–32mm drier; range 0–49mm drier; Table 5). For soils where depth was restricted, including Harden (restricted at 1.6 m), the soil was up to 4mm drier (site ranges; 0–11mm). Similarly, in a system where crops were sown early, mean PAW at sowing was 7–21mm drier on unconstrained soils and 2–6mm drier on soils with root constraints. The combination of early cultivars with modified root systems resulted in even drier soil at sowing (23–44mm and 3–8mm on unconstrained and constrained soils, respectively; Table 5).

**Table 5.** Mean plant available water (PAW) at sowing (mm) at eight sites in annually reset and continuous simulations for the standard cultivar sown in the conventional window and the reduction in PAW at sowing due to the legacy of either modified root systems (Mod), early sowing of a longer-season cultivar, or a combination of both. Values are mean of 100 years of simulation

Location^a^	PAW at sowing (mm) – standard cultivar, conventional sowing		Reduction in PAW at sowing (mm) (relative to standard cultivar in continuous simulation)		
	Reset	Continuous	Mod	Early	Early, mod
Wongan Hills, WA	103	101	32	21	44
Dalby, QLD	217	221	26	16	37
Cootamundra, NSW	182	192	17	7	23
Ardlethan, NSW	146	162	26	11	32
Harden, NSW	113	125	4	3	8
Paskeville, SA	49	50	3	6	8
Esperance, WA	58	57	1	2	3
Birchip, VIC	57	61	4	3	7

^a^ Australian states listed are: QLD, Queensland; NSW, New South Wales; SA, South Australia; VIC, Victoria; WA, Western Australia.

### Grain yield

**Fig. 5.**
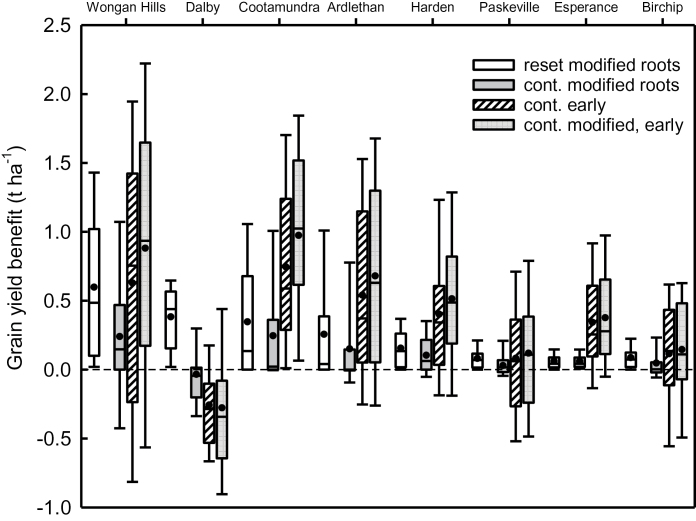
Box plots of simulated yield benefit of cultivars with either modified root systems and/or were sown early relative to the standard cultivar sown in the conventional window at eight sites varying in climate and soil type. Simulations were either reset annually (white) or run continuously (shaded) so that the legacy of crop history affected soil water content. Median (black line), mean (circle), 25th and 75th percentile (box), 10th and 90th percentile (whisker) are presented for 100 years of simulation.

Mean grain yields for standard cultivars sown in the conventional window ranged from 3.1 to 5.7 t ha^−1^ across the eight sites, with higher yields occurring at sites with more rainfall and deeper soils (Table 6). In the reset simulation, modified root systems led to a mean yield increase of 0.1–0.6 t ha^−1^, which varied with site, while yield benefits were smaller (−0.03–0.24 t ha^−1^) in the continuous simulation. At Dalby, there was a mean yield loss in the continuous simulation (0.03 t ha^−1^ loss compared to a 0.38 t ha^−1^ benefit in the annually reset simulation). For all sites the reduced benefit of modified root systems was associated with increased risk of yield loss in some years in the continuous simulation compared to the annually reset simulation where no downside risk was predicted (Fig. 5).

**Table 6.** Mean grain yield and yield benefit (t ha^−1^) of modified root systems and/or earlier sowing of a longer-season cultivar, or a combination of both for 100 years of simulation at eight sitesYield benefit is the difference between grain yield of cultivars with standard roots, sown in the conventional window.

Location^a^	Annual reset				Continuous simulation			
	Mean yield(t ha^−1^)	Mean yield benefit (t ha^−1^)			Mean yield (t ha^−1^)	Mean yield benefit (t ha^−1^)		
	Standard	Mod	Early	Early, mod	Standard	Mod	Early	Early, mod
Wongan Hills, WA	4.02	0.60	0.78	1.39	4.01	0.24	0.63	0.88
Dalby, QLD	3.48	0.38	−0.08	0.43	3.73	−0.03	−0.26	−0.28
Cootamundra, NSW	5.66	0.35	0.65	1.00	5.66	0.25	0.75	0.97
Ardlethan, NSW	4.69	0.26	0.37	0.69	4.90	0.15	0.54	0.68
Harden, NSW	5.59	0.16	0.37	0.52	5.60	0.10	0.40	0.51
Paskeville, SA	3.76	0.08	0.03	0.09	3.73	0.03	0.08	0.12
Esperance, WA	3.89	0.06	0.40	0.43	3.91	0.06	0.34	0.38
Birchip, VIC	3.12	0.09	0.03	0.11	3.08	0.05	0.12	0.15

^a^ Australian states listed are: QLD, Queensland; NSW, New South Wales; SA, South Australia; VIC, Victoria; WA, Western Australia.

In the continuous simulation, benefits of early sowing were greater than those of modified root systems at every site (0.1–0.8 t ha^−1^), except at Dalby where on average a greater yield loss was predicted (−0.26 t ha^−1^; Table 6). In general, annual resetting resulted in similar or smaller mean annual benefit from early sowing than continuous simulation when crops were sown early (with or without modified root systems). The mean yield benefit from the combination of root modification and early-sown longer-season cultivars was equivalent to the sum of the two individual components in most cases (Table 6). Variability in yield benefit from early sowing was much greater than was predicted for modified root systems (Fig. 5). The range was largest at Wongan Hills, where yield benefits from early sowing ranged from a reduction of 0.8 t ha^−1^ to a benefit of 2.0 t ha^−1^. The combination of modified root systems with early sowing resulted in a small further increase in variability.

**Fig. 6.**
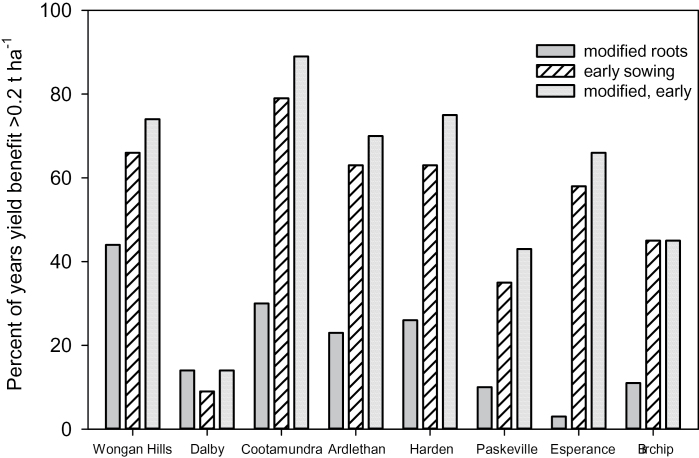
Percentage of years when yield benefit is greater than 0.2 t/ha at eight sites for cultivars which had modified root systems, longer vegetative period and were sown earlier, or a combination of both. Results are derived from 100 years of continuous simulation without resetting of soil water profile.

In the annual reset simulations the proportion of years with a significant yield benefit (defined here as >0.2 t ha^−1^) was similar to that reported by Lilley and Kirkegaard (2011) at common sites (data not shown). For continuous simulations, the deepest soil at Wongan Hills had the highest proportion of years with a significant yield benefit from modified root systems (44%; Fig. 6). Other sites with deep soils had a significant yield benefit in fewer years (23–30%) and shallow soils had the smallest frequency of benefit (3–11%; Fig. 6).

Early sowing resulted in a much greater frequency of significant yield benefits than modified root systems at all sites except Dalby (35–79%) (Fig. 6). Notably, early sowing produced significant yield benefits in 35–58% of years on shallow soils. A further increase (up to 11%) in frequency of yield benefits >0.2 t ha^−1^ was reported when root system modification was combined with early sowing. At Dalby, where the mean response to early sowing was negative, a yield response >0.2 t ha^−1^ was reported in only 9% of years. Modified root systems provided a yield benefit >0.2 t ha^−1^ in 14% of years for both early and conventional sowing windows at Dalby (Fig. 6).

## Discussion

Our study suggests that previous investigations may have significantly overestimated the value of deep roots in Australian dryland farming systems by ignoring legacy effects. Previous studies (Dreccer *et al.*, 2002; King *et al.*, 2003; Manschadi *et al.*, 2006; Semenov *et al.*, 2009; Lilley and Kirkegaard, 2011) all involved annual resetting of soil water and we have shown using continuous simulation that the legacy of drier soils caused by more effective root systems will reduce predicted yield benefits to the subsequent crop in many seasons. At sites with shallower soils, which make up a significant area of the Australian cropping zone, the predicted benefits of more efficient root systems were negligible, while earlier sowing of slower-maturing crops delivered yield benefits on all of the soil types considered in Australia’s southern cropping zone.

### Benefits of root modification

Our current analysis showed a similar range in yields (3.5–5.7 t ha^−1^) and yield benefits from root modification (0.2–0.6 t ha^−1^) on the same deep soil sites (with annual reset) as the previous study. The yield benefit was attributed to 0.25–0.34 m deeper roots and a 14–29mm increase in water uptake. Simulation studies of Manschadi *et al.* (2006) in the northern cropping zone also reported a similar range of yield benefits when soils were one-third full at sowing.

For the new, shallow soil sites, modified root systems made little difference to mean root depth (up to 0.04 m deeper), with an extra 4mm of water taken up and a smaller mean yield benefit than for deep soil (~0.1 t ha^−1^). At Birchip and Paskeville, the benefits of root modification were small, since most of the soil water was extracted by the standard cultivar and there was no additional water available for uptake by more efficient roots (see median soil water content at maturity, Fig. 1). In addition, two factors constrained root depth at Birchip. Firstly, high boron content slowed root penetration, and secondly the low and variable rainfall (mean 365mm) combined with the large water holding capacity in the surface layers of this soil (Fig. 1), meant that water often did not penetrate deeply, and dry soil limited root penetration. At Esperance, which had a relatively high rainfall and a soil with a low water holding capacity, the profile filled frequently and adequate soil water was available within the 1 m root zone, so that soil water supply generally met demand from shoots, and water uptake did not limit growth of the standard cultivar. This is confirmed by the relatively high median soil water content at harvest for standard roots (Fig 1) and high frequency (99%) of years that roots reached the maximum depth. Comparison of Ardlethan and Cootamundra, which had an identical soil type, shows that at the drier site (Ardlethan), the profile filled less frequently and average rooting depth was 0.25 m shallower due to more frequent limitation to root penetration of dry soil, as reported by Lilley and Kirkegaard (2011).

### Legacy effects

Our analysis showed that increased water extraction by modified root systems leaves the soil in a drier state in most seasons, and where the soil does not refill this had an additional impact on the subsequent crop. This finding is consistent with experimental evidence from Kirkegaard and Ryan (2014), who showed large and significant impacts of cropping history on wheat yield (0.6–0.9 t ha^−1^), which persisted for three to four years in semi-arid cropping environments of Australia and particularly in seasons with below average rainfall. Angus *et al.* (2015) also reviewed field experiments in Australia and Sweden and showed that a range of crop species can have an impact on the yield of subsequent wheat crops and these effects can last more than one season, depending on intervening rainfall patterns. In the previous study (Lilley and Kirkegaard 2011), this legacy effect was demonstrated by comparing root exploration following either an annual crop or lucerne which had dried the soil to a much greater extent. For example, at Ardlethan where fallow rainfall was low (mean 187, range 45–450mm) benefits of modified root systems were observed less frequently following lucerne than an annual crop. This new study focussed on benefits of root modification in continuous crop simulations where the legacy of previous crops and seasons affects current crops, as happens in reality.

Continuous simulation showed that in a cropping sequence, the legacy of modified root systems meant that the profile was 17–32mm drier at sowing of the subsequent crop while in reset simulations no such impact is accounted for. The legacy of dry soil varied seasonally and for deep soils, the increased frequency of dry soil decreased the mean rooting depth of subsequent crops and hence the root penetration benefit of improved root vigour. As a consequence of reduced soil water availability and reduced root penetration, the benefit in water uptake from modified root systems was smaller in continuous compared to reset simulations. The ‘dry soil legacy’ reduced the mean predicted yield benefit of modified root systems in the continuous simulations to 0–0.2 t ha^−1^ (range −0.4–1.1 t ha^−1^) compared to the annual reset simulations (mean 0.1–0.6; range 0–1.4 t ha^−1^) as reported in previous studies.

At Dalby, the legacy effect of soil drying was so large that in 67% of years rooting depth of the modified cultivar was shallower than the standard cultivar (mean reduction 0.06 m, range +0.23 m to –0.43 m; Fig. 3). The drier soil and reduced rooting depth resulted in a reduction in water uptake by the crop in more than 50% of years, and average additional uptake due to root modification was much less in continuous (mean of 3mm, median of −1mm) than reset simulations (mean and median of 15mm). The reduced water uptake was related to a reduction in grain yield in around 75% of years, and a yield benefit >0.2 t ha^−1^ was predicted in only 14% of years. In the northern cropping zone, Hochman *et al.* (2014) showed that decisions in crop sequence management are based on soil water content as a strategy for managing legacies of previous crops and seasonal conditions. The cropping system in this summer-dominant rainfall zone differs from those in southern Australia as a range of summer and winter crops are well adapted to the region, while southern Australia is limited to winter cropping (Hunt *et al.*, 2013; Hochman *et al.*, 2014) In reality, soil which is too dry to support a crop would be left fallow to accumulate soil water for a subsequent summer or winter crop and growers need to be mindful of cultivar and species choices which leave a legacy of dry soil.

On shallow soils (~1 m), rooting depth was restricted by other soil constraints, discussed above, and root system modification had little effect on final root depth. The effect on extra water uptake was also small, although there was an increase in variability and a small decrease in mean uptake at Paskeville and Birchip. Consequently, there was not a significant legacy effect on PAW at sowing (1–4mm) as roots of the standard cultivar fully dry the soil in most years and modified root systems provided little additional extraction capacity.

In semi-arid farming systems such as Australia and north Africa, where the soil profile does not refill in many seasons (Cooper *et al.* 1987), analyses that involve annual resetting of soil water content have typically overestimated the benefit of more extensive root systems. For example, the analysis of Lilley and Kirkegaard (2011) reported that there was no downside risk of introducing modified root systems, however in this study the legacy of previous crops with modified root systems resulted in negative effects on yield in 25% of years (Wongan Hills, Ardlethan, Harden, Paskeville and Birchip) and in 75% of years at Dalby (Fig. 5). These negative effects were rare at the higher rainfall sites at Esperance and Cootamundra where the profile refilled more frequently.

### Deep vs shallow soils

Kirkegaard *et al.* (2007) showed that because deep water is accessed late in crop growth it is particularly valuable as it is used during the grain-filling period and contributes efficiently to grain yield. Much of the previous work on the value of improved root systems focused on deeper soils where there is potential to increase the depth of rooting (Manschadi *et al.*, 2006; King *et al.*, 2003; Lilley and Kirkegaard 2011). However, in Australia much of the cropping zone has soils with constraints below 0.5–1.0 m which reduce or prevent root exploration (salinity, sodicity, acidity, alkalinity, and toxicities or deficiencies of micronutrients; Dolling *et al.* 2001; Passioura, 2002; Adcock *et al.*, 2007; McDonald *et al.*, 2012). Two simulation studies (Dreccer *et al.*, 2002; Semenov *et al.*, 2009) which considered benefits to wheat yield of increased uptake efficiency in shallow soils found that the yield benefit was small, despite optimal water availability due to a full profile at sowing. Our new study also showed that on shallow soils there was no rooting depth benefit. Increased efficiency of uptake resulted in a small additional extraction (2–4mm for soils ~ 1 m deep) and yield benefits were generally small and infrequent (benefits >0.2 t ha^−1^ in 3 to 11% of years; Fig. 6). At Harden, where the soil was not shallow, but depth was restricted to 1.6 m, significant extra uptake occurred (mean 7mm) and a yield benefit >0.2 t ha^−1^ was reported in 26% of years. Seasonal variability in the size of the yield benefit from modified root systems was much greater at sites with deep soil since water storage was also variable, while the benefit on shallow soils was consistently low due to the limited water holding capacity (Fig. 5).

### Benefits of early sowing

Changing the duration of the vegetative period affects the final rooting depth of wheat as root growth ceases around the time that grainfilling commences, due to increased demand for assimilate from the developing grain (Gregory 2006; Kirkegaard and Lilley, 2007; Thorup-Kristensen *et al.*, 2009). Simulation and field studies by Kirkegaard and Hunt (2010) and Kirkegaard *et al.* (2014), have recently shown that earlier sowing of wheat increases potential crop yield, provided that flowering remains in the optimal window to avoid frost. The early sowing of a longer duration cultivar in this study resulted in a 3-week longer period of downward root growth and similar climatic conditions during grain filling as flowering occurred at a similar time to the conventionally sown cultivar (mean difference 1–2 d).

The mean legacy of drier soil from early sowing was smaller than from modified root systems (on deep soils; 10–15mm wetter after early sowing), while the mean yield benefit of early sowing was always greater than for modified root systems at southern sites. The mean yield benefit of early sowing over the conventional sowing date ranged from 0.54 t ha^−1^ at Ardlethan to 0.75 t ha^−1^ at Cootamundra (deep soils). In southern cropping zones, Kirkegaard *et al.* (2014) attributed much of the early sowing benefit to a longer period of water extraction, resulting in greater total transpiration, and less soil evaporation on an annual basis, increasing the seasonal water use efficiency. Although early sowing increased mean water uptake at Dalby by 14mm, the mean effect of early sowing at that site was a reduction in grain yield, with yield benefits >0.2 t ha^−1^ reported in only 9% of years (Fig. 6). Small negative effects of early sowing on yield were also reported for the northern cropping zone by Hochman *et al.* (2014).

For restricted soils, including Harden, the extra water extraction for early-sown crops was also large (mean; 22–26mm). This extra uptake was achieved through longer season length and greater rainfall capture rather than more extensive soil exploration and there was little effect on PAW at maturity (data not shown). Consequently, the soil water legacy for the following crop was also small, (mean; 2–6mm). Notably, for shallow soils the yield benefit from early sowing was much greater (>0.2 t ha^−1^ in 35–48% of years) than from root modification (3–11% of years). The yield benefit from early sowing was particularly high at Esperance, where the profile had ample water throughout the crop growth period in many years, so that a longer growth period allowed increased uptake and a yield benefit >0. 2 t ha^−1^ in 58% of years. Seymour *et al.* (2015) and Bell *et al.* (2015) have shown that early sowing is well suited to this region due to the high rainfall, and frequent opportunities to sow early.

Manschadi *et al.* (2006) and Semenov *et al.* (2009) discussed the trade-off between more rapid water-use in the early part of the season in anticipation of late season rainfall vs. conserving water for use during grainfilling when the benefit to grain yield is known to be high. Our results suggest that on deep soils the majority (66–80%) of the additional water uptake by modified root systems occurred post-anthesis, while on shallow soils this was 45–67% although the difference in total uptake was very small (mean; 2–7mm). In contrast to modified root systems, mean post-anthesis uptake in early-sown crops decreased by 5–11mm at all sites except Wongan Hills where deep soil water supply was generally greater than demand. While early-sown crops used more water over the season, the post-anthesis water use was less at most sites because these crops had depleted the available water supply by anthesis. This phenomenon of increased total water use, but decreased post-anthesis water uptake has been observed in several experimental studies in south-eastern Australia (James Hunt, unpublished.)

The benefits to water extraction and yield from early-sown, slow-maturing cultivars and modified root systems appeared to be additive, with the combination resulting in a small further yield benefit beyond that of early sowing (further 0.1–0.3 t ha^−1^ on deep soils and 0.03 t ha^−1^ on shallow soils). However, there was a greater legacy effect, with mean PAW at sowing reduced by 23–44mm on deep soil sites and 3–8mm on shallow soil sites.

### Implications for improved productivity in future rain-fed environments

The current analysis has been conducted on the historical climate record, however the future climate is unlikely to be the same, and variability and production risk is expected to increase (Howden *et al.*, 2007). In southern Australia, a decrease in growing season rainfall has also been observed (Pook *et al.*, 2009; Cai *et al.*, 2012), making the efficient use of carry-over soil water and fallow rainfall an important consideration (Hunt *et al.*, 2013). This will exacerbate variability in refilling of the soil after a crop and potentially increase the significance of soil water legacies. Kirkegaard and Hunt (2010) showed benefits of early sowing are likely to persist under climate change where weather will generally be hotter, drier and more variable, however genetic differences in roots are likely to be more problematic due to more variable soil refilling.

These findings support and extend the work of Lilley and Kirkegaard (2007, 2011), who showed that a range of management factors such as fallow weed control, preceding crop legacy and timely sowing often exceeded or overrode the impact of root modification on yield by influencing the depth of profile wetting and duration of root descent. Though our continuous simulation better matches reality, the simulation rules were fixed, where as in practice farmers can manage the crop sequence dynamically, electing to sow crops that have a smaller water requirement following crops and seasons which leave dry profiles (Hochman *et al.*, 2014). Inclusion of a legume or green manure crop can preserve water and has disease break, weed control and nitrogen-saving benefits to the farming system, but must be profitable for such choices to be made (Hochman *et al.*, 2014; Angus *et al.*, 2015). Crop choice is ultimately driven by current soil water status, seasonal forecasts (weather and market), and paddock history in relation to disease and weed break rotations and market value of the crop (Moeller *et al.*, 2009; Oliver *et al.*, 2010; Hunt *et al.*, 2013; Hochman *et al.*, 2014). Thus, annual crops with deeper and more effective root systems can be used tactically in crop sequences to capture benefits from deep water when it is available. Information from soil moisture sensors and/or simple models of soil water availability (e.g. HOWWET?; Dimes *et al.* 1996) would assist farmers to manage the sowing window in a more flexible way. Availability of cultivars that have a wide sowing window yet flower in the optimal period to minimize frost and heat risk will also improve options for earlier sowing (Kirkegaard *et al.* 2014; Richards *et al.* 2014; Hunt *et al.* 2015). This analysis indicated that in some circumstances a yield loss is associated with more effective root systems so it is important to consider when it is appropriate to include crops with more extensive root systems in the rotation sequence.

## Conclusion

More extensive root systems are valuable for acquiring resources to increase crop yield, but create a legacy of drier soil for subsequent crops, which can reduce the predicted long-term system benefit at some sites. At sites with shallower soils, which make up a significant area of the Australian cropping zone, the benefits of more extensive root systems were negligible. On all soil types in Australia’s southern cropping zone, earlier sowing of slower-maturing crops increased average yield. Managing risk associated with more variable future climate will require species and cultivar choices in sequences that optimize use of the available soil water. Wheat cultivars with deeper and more efficient root systems will need to be used tactically to optimize overall system benefits.

## Supplementary data

Supplementary data are available at *JXB* online.

Table S1. Values of several soil characteristics and APSIM parameters (defined in Keating *et al.*, 2003) used in the simulation studies.

Supplementary Data
