# Supplementary material for: Farming system context drives the value of deep wheat roots in semi-arid environments
Source: J Exp Bot. 2016 Mar 14;67(12):3665–81. doi: 10.1093/jxb/erw093 (PMC4896360; doi:10.1093/jxb/erw093)
Supplement: Supplementary Data [file supp_67_12_3665__index.html]

Farming system context drives the value of deep wheat roots in semi-arid environments — Farming system context drives the value of deep wheat roots in semi-arid environments — Supplementary Data 

# Farming system context drives the value of deep wheat roots in semi-arid environments

## Supplementary Data

Data files

- supplementary\_table\_S1.pdf - Supplementary Data
