# Supplementary material for: Farming system context drives the value of deep wheat roots in semi-arid environments
Source: J Exp Bot. 2016 Mar 14;67(12):3665–81. doi: 10.1093/jxb/erw093 (PMC4896360; doi:10.1093/jxb/erw093)
Supplement: Supplementary Data [file supp_erw093_supplementary_table_S1.pdf]

Supplementary Table 1. Values of several soil characteristics and APSIM parameters (defined in Keating *et al.,* 2003) used in the simulation studies.

Location [A]	APSIM soil description	diffus_const, diffus_slope [B]	U, Cona [C]	Total C (0-0.1 m) (%)	C:N ratio (0-0.1 m)	BOM [D] station number
Wongan Hills, WA	Buntine No 424 [E]	220 ,22	2, 2 (Apr-Nov)<br>6, 3.5 (Dec-Mar)	0.52	13	8138
Dalby, QLD	Macalister No 026 [E]	40, 16	4, 2.5 (Apr-Oct)<br>6, 3.5 (Nov-Mar)	1.04	12	41023
Cootamundra, NSW<br>Ardlethan, NSW	Lilley and Kirkegaard (2007)	44, 16	2, 2 (May-Oct)<br>6, 3.5 (Nov-Apr)	0.83	8	C: 73016<br>A: 74000
Harden, NSW	Lilley *et al*. (2004)	88, 35.4	2, 2 (May-Oct)<br>6 ,3.5 (Nov-Apr)	1.30	10.8	73009
Paskeville, SA	Kulpara No CY024 [E]	88, 35	2, 2 (Apr-Oct)<br>6, 3.5 (Nov-Mar)	1.40	12.5	22012
Esperance, WA	Gibson No 455 [E]	250, 25	2, 2 (Apr-Oct)<br>6, 3.5 (Nov-Mar)	0.90	14.5	9631
Birchip, VIC	Jil Jil No 727 [E]	40, 16	2, 2 (Apr-Oct)<br>6, 3.5 (Nov-Mar)	1.30	12	77007

[A] Australian states listed are: QLD – Queensland; NSW – New South Wales; VIC – Victoria; WA – Western Australia.
[B]Diffus_const and diffus_slope are parameters defining diffusivity of unsaturated water flow between soil layers.
[C]U and cona are parameters of a two stage soil evaporation rate.
[D]BOM – Australian Bureau of Meteorology http://www.bom.gov.au
[A,B] Further details at https://www.apsim.info/Documentation/Model,CropandSoil/SoilModulesDocumentation/SoilWat.aspx
[E]Further details at https://www.apsim.info/Products/APSoil.aspx
